# Gene Delivery Technologies with Applications in Microalgal Genetic Engineering

**DOI:** 10.3390/biology10040265

**Published:** 2021-03-26

**Authors:** Sergio Gutiérrez, Kyle J. Lauersen

**Affiliations:** Biological and Environmental Sciences and Engineering Division, King Abdullah University of Science and Technology (KAUST), Thuwal 23955-6900, Saudi Arabia; sergio.gutierrezzapata@kaust.edu.sa

**Keywords:** microalgae, transformation, cyanobacteria, DNA

## Abstract

**Simple Summary:**

Microalgae and cyanobacteria are considered intriguing microbes for sustainable biotechnological production of a wide array of high-value metabolites from carbon dioxide and (sun)light. Mature genetic engineering concepts have only recently begun to emerge with the advent of customized DNA synthesis due to complicated genetics in these hosts. The delivery of heterologous DNA into cells is the first step in engineering concepts, yet is highly diverse in methodology, efficacy of expression cassette delivery, and applicability to variable organisms. This work overviews common and not-so common methodologies of DNA delivery, which may find use in engineering concepts for photosynthetic microbes.

**Abstract:**

Microalgae and cyanobacteria are photosynthetic microbes that can be grown with the simple inputs of water, carbon dioxide, (sun)light, and trace elements. Their engineering holds the promise of tailored bio-molecule production using sustainable, environmentally friendly waste carbon inputs. Although algal engineering examples are beginning to show maturity, severe limitations remain in the transformation of multigene expression cassettes into model species and DNA delivery into non-model hosts. This review highlights common and emerging DNA delivery methods used for other organisms that may find future applications in algal engineering.

## 1. Introduction

Microalgae and cyanobacteria are interesting study organisms capable of photosynthetic growth on carbon dioxide (CO_2_) as a sole carbon source. These organisms naturally synthesize different metabolites such as pigments, oils and lipids, sterols, starches, polysaccharides, and halogenated compounds, and algae are also amenable to contained, scalable growth in photobioreactors driven by (sun)light energy [1,2]. Genetic engineering of algae and cyanobacteria in the era of synthetic biology holds the promise of tailored production of novel and customized metabolites using sustainable waste CO_2_ as a feedstock. However, algae are a diverse and polyphyletic group of organisms which do not share close evolutionary relatedness and exhibit incredible variability in their genomes [3]. These features have hindered intensive molecular tool development except in a handful of model species, and their broader application to biotechnology has been slower compared to other hosts such as bacteria, yeast, plant, and mammalian cells [2,4].

The genomic diversity of algae necessitates customized molecular tools that work with genetic architecture of a specific host. Once these tools are produced by piecemeal cloning or complete DNA synthesis, the reliable introduction of foreign genetic material into cells is an essential prerequisite to biotechnological concepts [5,6,7]. DNA delivery methods result in stable chromosomal integration or episomal/plasmid extrachromosomal replication of foreign transgene expression elements [7,8,9]. Methodologies of DNA delivery and cell membrane/wall permeabilization vary according to the host organism and target cellular compartment (organellar or nuclear). For example, cyanobacteria will natively take up DNA from their environment without the need for manipulation, while many eukaryotic algae maintain cell walls that necessitate more aggressive delivery methods [10,11]. 

**Figure 1:**
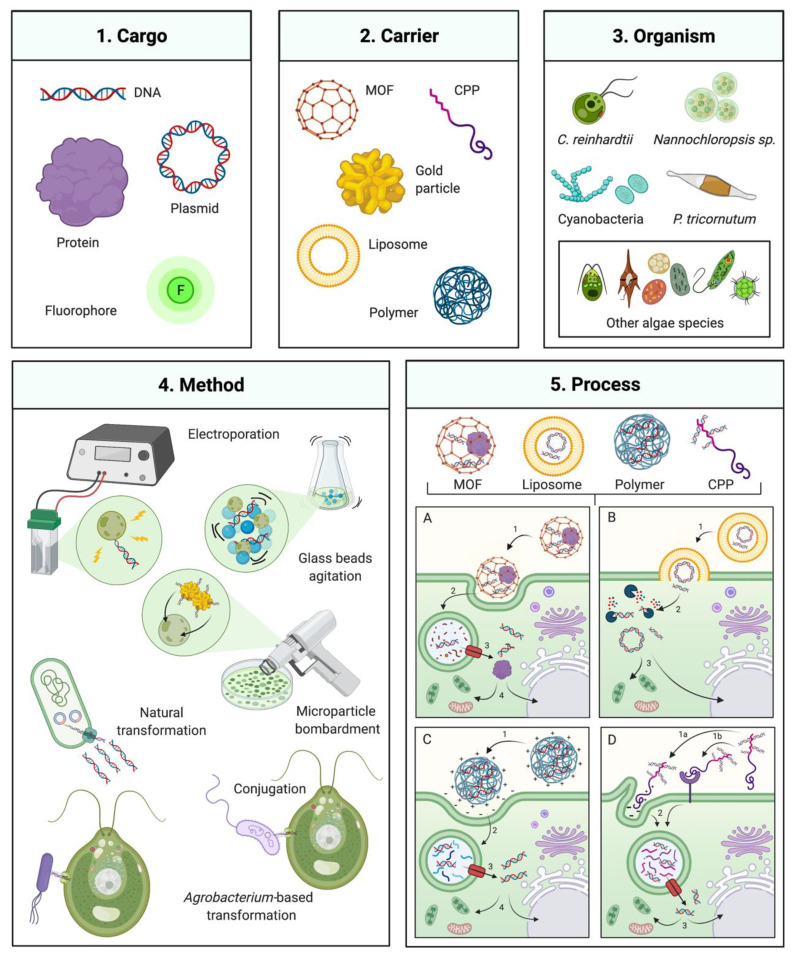
Gene delivery technologies for microalgae engineering. Overview of several available transformation technologies which have been or could be applied to algal engineering. (**1**) and (**2**) Several carriers that can mediate transformation of a DNA, protein, or chemical cargo (left panel) MOF: metal-organic framework, CPP: Cell-penetrating peptide. (**3**) Algal species which transformation has been commonly employed are depicted on the upper right: *C. reinhardtii, Nannochloropsis sp.,* and *P. tricornutum*, including various cyanobacteria and other algal species as described in the manuscript. (**4**) Classical transformation methods are presented in the bottom left panel. (**5**) Process of transformation mediated by different DNA carriers and their interaction with the cell for delivery of the respective cargo. (**5A**) MOF-DNA, (**5A1**) recognition and cell uptake by endocytosis, (**5A2**) internalization and fusion with phagolysosome, **3** phagolysosome escape, **4** transport to nucleus, chloroplast or mitochondria; (**5B**) Liposome-DNA, (**5B1**) lyposome integration with cell membrane, (**5B2**) cargo (DNA or protein) exposed in cytoplasm and potential enzymatic degradation, **3** intact cargo transport to nucleus, mitochondria or chloroplast; (**5C**) Polymer-DNA, (**5C1**) cell recognition of polymer nanoparticle, charge interaction and cell uptake by endocytosis, (**5C2**) internalization and fusion with phagolysosome, (**5C3**) phagolysosome escape, (**5C4**) transport to nucleus, chloroplast or mitochondria; (**5D**) CPP-DNA, **1** cell recognition of CPP, by charge interaction **(5D1a)** or receptor recognition (**5D1b**) and cell uptake by endocytosis, (**5D2**) internalization and fusion with phagolysosome, (**5D3**) phagolysosome escape, (**5D4**) Transport to nucleus, chloroplast or mitochondria. Created with BioRender.com.

Foreign DNA delivery can be achieved by mechanical agitation, surfactant permeabilization, electroporation, particle bombardment, and bacterial DNA transfer (conjugation or *Agrobacterium tumefaciens*-mediated) [9,12,13,14,15]. In addition to these classical methods, emerging techniques used in other cell systems that have not been widely applied to algae may potentially improve DNA delivery (Figure 1). Strategies based on cell penetrating peptides (CPP), polymers, metal-organic frameworks, nanoparticles, and liposomes have all been demonstrated in non-algal hosts [16,17,18]. This review examines recent progress in gene delivery methodologies and discusses their technical aspects, advantages, limitations, and potential in the context of algal biotechnology.

## 2. Traditional Algal Transformation Techniques

### 2.1. Agitation of Cells in the Presence of DNA and Non-Ionic Surfactants

The agitation of algal cells in the presence of glass beads, polyethylene glycol (PEG), and foreign DNA has become a standard protocol for the delivery of foreign DNA into the model green microalgae *C. reinhardtii* and other species as *Cyanidioschyzon merolae, Chlorella vulgaris,* and *Dunaliella salina* [14,19,20,21]. This protocol has been adopted for nuclear and chloroplast transformation and can be applied to cell wall deficient cell lines or those with a cell wall when chemo/enzymatic removal is performed before transformation (as with *Chlorella* strains) [13,14,15,22,23,24]. Agitation-based transformation is achieved by agitation (vortexing) of cells in the presence of a physical agitator (glass beads), a non-ionic surfactant (i.e., polyethylene glycol (PEG)), and DNA [14,25]. Transformation efficiency depends on many factors such as cell size, presence or absence of a cell wall, duration of agitation, velocity, the concentration of surfactant, and the use of linear or circular DNA [26]. The frequency of DNA integration into the *C. reinhardtii* genome with glass bead agitation has been reported at ~10^3^ transformants μg of DNA^-1^ with ~10^8^ cells mL^−1^ starting cell concentration [14]. Reports have shown that numerous other non-reactive materials, such as silicon carbide whiskers can also serve this purpose [27]. Agitation-based transformation protocols are advantageous as they do not require specialized equipment, and are inexpensive and relatively fast [14,15]. 

A significant limitation of the technique is the requirement of cell wall removal, which can be achieved with the autolysin protein of *C. reinhardtii* for itself, or other enzyme cocktails (e.g., cellulase, macerase, pectinase, and hemicellulose) for other hosts [14,19,28,29,30,31,32]. Reports have shown that cell-wall free protoplasts of *Chlorella ellipsoidea* and the naturally cell wall deficient *C. merolae* have been transformed using DNA and PEG without mechanical agitation, suggesting that surfactant mediated permeabilization is sufficient to enable DNA uptake [33]. However, the main drawback of agitation-based transformation techniques is the low transformation efficiency [34]. This has led to development of other more efficient strategies. 

### 2.2. Electroporation

DNA transformation by electroporation is as an alternative method in numerous algae and pioneered in *C. reinhardtii* [34]. This technique uses electrodes to generate voltage differential across the cell membrane, temporarily disturbing the phospholipid bilayer, allowing molecules to pass into the cell [35,36]. Transformation efficiencies from electroporation vary according to factors such as field-strength, pulse length, ionic strength of medium composition, temperature, cell membrane characteristics, the species used, its molecular toolkit, and the presence of cell-wall [36,37]. Electroporation can increase transformation efficiency up to 100-fold over agitation and is not affected by the cell wall. Fewer sequence deletions have been observed at the genomic insertion sites than those seen from agitation transformation [34,37,38]. Electroporation has been shown to enable the transformation of cell-wall containing algae and cyanobacteria such as *Monoraphidium neglectum*, *Nannochloropsis sp.*, *Phaeodactylum tricornutum*, *C. reinhardtii, Anabaena, and Nostoc punctiforme* [34,35,39,40,41,42,43,44]. Electroporation is a useful technique for delivering DNA, RNA, proteins, nucleotides, and dyes into cells [34,35,45]. Electroporation is a rapid protocol and requires equipment which is often present in microbial laboratories to transform bacterial cells [37,46]. 

Electroporation efficiencies were increased by considering the specific mitotic phase of *C. reinhardtii* growth [47]. Synchronization of *C. reinhardtii* cultures has been shown to increase transformation efficiency after three days. *C. reinhardtii* culture synchronization using 28 °C during the light phase and 18 °C in the dark phase was also shown to enable increased homologous recombination efficiencies specifically at 12 hours after illumination initiation [48]. Digital microfluidics (DME) have also been used to optimize electroporation protocols, using a fluid mixture of cell/DNA droplets encapsulated in biocompatible oil, and electric pulses applied from an array of microelectrodes to the droplets [49,50]. DME showed an efficiency of 2.5 × 10^4^ of *C. reinhardtii* mutants per μg of DNA without cell wall removal with an initial concentration of 2 × 10^6^ cells mL^−1^ algal cells, and 1 μg DNA [49]. A square-electric-pulse electroporation technique has also been reported with efficiencies of 6 × 10^3^ *C. reinhardtii* transformants per μg of DNA with 1.5 × 10^7^ cells mL^−1^ initial cell density 0.1 μg of DNA [47]. However, this technique has not been broadly adopted, as it requires specialized equipment. It was described in *N. limnetica*, which contains a rigid cell wall, that treatment with 10 mM lithium acetate and 3 mM dithiothreitol (DTT) before electroporation improved transformation efficiency by increasing cell wall permeability, resulting in 1.1 × 10^7^ transformants per μg of DNA with an initial concentration of 3.3 × 10^9^ cells mL^−1^ and 4 μg of DNA [40]. 

### 2.3. Microparticle Bombardment

Microparticle bombardment is one of the most versatile gene delivery methods due to its ability to transform the nucleus, mitochondria, or chloroplast genomes and even transform cells containing cell walls [13,51,52,53]. Microparticle bombardment is based on accelerated non-reactive metal (gold or tungsten) micro-projectiles coated with DNA being shot at and colliding with target cells [1,51]. The impact of these particles allows them to penetrate the cells and deliver foreign DNA. Transformation efficiency depends on the starting cell density, target organelle, selection efficiency, the number of DNA-coated particles, the DNA concentration on each particle, the kinetic energy of the particles, temperature, and ability of the cell to regenerate after particle damage [54]. 

The most common use for this technique is the transformation of chloroplast genomes, as it enables penetration of DNA through multiple membrane layers, but it has also recently been used to transform circular plasmids into the nucleus of the red alga *Porphyridium purpureum* [23,51,55]. Unlike agitation, but similar to electroporation, cell-wall presence does not affect transformation efficiency; however, cell viability can be disturbed if many micro-projectiles are used [51,56]. The efficiency of nuclear transformation in *C. reinhardtii* has been as low as 15 transformants per μg of DNA, with ~10^7^ cells mL^−1^ starting cell concentration and 0.8 μg of DNA [13]. Efficiencies reported in *D. salina, Volvox carteri* and *P. tricornutum* are 2.5 × 10^−5^, 1.7 × 10^2^, and 1.0 × 10^2^ transformants per μg of DNA (0.1–0.7 μg of DNA), respectively, with 10^5^–10^7^ cells mL^−1^ as starting cell density [57,58,59]. However, it was reported that in *C. reinhardtii,* transformation efficiency could be improved when smaller particles are used (0.6 µm vs. 1.0 µm) [60]. 

Microparticle bombardment has also been described for the delivery of 24–68 kDa proteins, a technique called proteolistics. This approach is a simple physical deposition of target protein onto delivery substrate which is then used as other microprojectiles [61]. The application was shown to deliver Cas9–gRNA ribonucleoprotein (RNP) into *P. tricornutum*, where knock out of the *PtUMPS* and *PtAPT* genes led to 5-fluoroorotic acid (5-FOA) and 2-fluoroadenine (2-FA) resistance at an efficiency of ~10^−6^ per 10^6^ cells mL^−1^ [62]. This technique was also found to be suitable for multiplexing mutations [62] and could become a powerful strategy for generating targeted knockouts in this and other diatoms/algae.

One disadvantage of bombardment is the requirement of gene-gun infrastructure as well as reagents and equipment. Transformation parameters must also be optimized for every alga and target cell compartment/genome [9,63]. One thing that must be considered is the potential post-transcriptional gene silencing (PTGS) due to the relatively high copy number of transgenes integrated into the genome [53,64].

## 3. Natural Transformation, Bacterial Conjugation, and *Agrobacterium*-Mediated Transformation

### 3.1. Natural Transformation

The natural uptake of DNA from the surrounding environment without surfactants is a process found in cyanobacteria species [65,66]. This natural transformation capacity was exploited in 1970 as a simple tool for cyanobacterial genetic engineering [67]. Biotechnologically relevant species such as *Synechocystis* PCC 7942, *Synechococcus* PCC 7002, and *Thermosynechococcus elongatus* BP-1 are naturally transformable [65,67,68,69]. However, the detailed mechanism of natural transformation remains unclear, although pili type IV and secretion system type II have been shown to play roles in this process [70,71,72,73]. Natural transformation functions together with replicative and integrative plasmids, which differ only in their homologous recombination regions or replicative elements. Transformation efficiency relies on cyanobacterial physical and chemical features (e.g., polysaccharides), culture state (i.e., mid-exponential growth), and the use of DNase inhibitors (i.e., EDTA) [74,75]. Foreign DNA characteristics such as concentration, length, and single or double-stranded state play a role in the number of transformants obtained [66,76,77]. Natural transformation provides the most practical and straightforward method for transformation; however, it is limited to species in which this a native feature [74].

### 3.2. Bacterial Conjugation

Conjugation is based on the ability of bacteria to share genetic information by exchanging plasmids through pili. This natural process can be employed using either a double or triple vector approach combining shuttle, conjugative, and helper plasmids. The shuttle and helper plasmids mediate the transfer of a conjugative plasmid between *E. coli* and the target host while the helper plasmid aids transformation by preventing vector degradation by endogenous restriction systems; it codes for DNA methylases (Aval, AvaW, and AvaIII) that prevent target host restriction enzyme recognition [78,79]. The helper vector contains an oriT-region, *bom*-site, and *mob* genes that encode a nickase and enable conjugation [80,81]. Bacterial conjugation has been used commonly in cyanobacterial species such as *Synechococcus, Prochlorococcus, N. punctiforme, Anabaena*, and *Synechocystis* sp. and recently in eukaryotic algae such as *P. tricornutum, Thalassiosira pseudonana, Acutodesmus obliquus,* and *Neochloris oleoabundans* [79,81,82,83,84,85,86,87,88]. The effectiveness of transformation by conjugation is based on the capability of the algal recipient strain to integrate and maintain the vector, either in the chromosome, in endogenous plasmids, or as an episomal plasmid [78,79,87,88]. 

Episomes, circular plasmids which self-replicate and do not integrate into the chromosome, can be efficient and straightforward vectors to enable transgene delivery of desired genetic elements between bacteria, cyanobacteria, and eukaryotes. Episomes avoid insertions and knock-out of non-targets and replicate independently from chromosomes [89,90,91]. Yeast centromere sequences (CEN/ARS) were found to act as autosomal replicating elements that allow stable maintenance of the circular episomal plasmid in diatoms [79,92]. Factors such as the growth phase of both target and donor organisms are crucial factors for proper conjugation [93]. Episomal vectors to transform microalgae via conjugation provide an efficient means of multi-gene pathway transfer due to stable self-replication of episomal vector and minimal possibility of positional or epigenetic effects [79]. Conjugation has the advantage over electroporation, microparticle bombardment, and glass-beads agitation to deliver larger DNA fragments [45]. The use of the conjugation-based method in *P. tricornutum, N. oceanica,* and *Neochloris oleoabundans* has been demonstrated to generate mutants with higher transformation efficiency compared to microparticle bombardment transformation: 500–1000 transformants/10^8^ cells/200 ng DNA (conjugation) compared to 5–25 transformants/10^8^ cells/2.5 µg DNA (bombardment) in *P. tricornutum* [87,94,95,96].

### 3.3. Agrobacterium-Mediated Transformation

The use of *A. tumefaciens* to transform plant cells relies on the natural infection process of the bacterium, which causes crown gall tumors on various plant species [97]. Natural tumor formation is a consequence of replicating a single-stranded copy of the transferred bacterial tumor-inducing (Ti) plasmid. The Ti plasmid and *A. tumefaciens* have been exploited since 1988 when it was found to allow transfer and permit stable integration of DNA fragments into a target higher plant genomes and has even been shown to transform mammalian cells [98,99,100]. The technique has the reported advantage of low gene rearrangements and foreign transcript silencing in plant cell lines [101,102].

*A. tumefaciens*-based transformation of algae has been reported in several species, although its use has not been wildly adopted. Reports exist of transformation by *Agrobacterium* method in *C. reinhardtii*, *H. lacustris*, *Chlorella sp., Dunaliella bardawil, Symbiodinium* sp., *Nannochloropsis* sp. and *Parachlorella kessleri* [98,103,104,105,106,107]. However, questions remain about how the bacterium, which evolved to infect plant cells, can infect evolutionarily distant algae and whether the infection requires specific recognition machinery on the target, or whether it is target independent. The standard protocol for *Agrobacterium*-mediated transformation is to mix target cell cultures with *Agrobacterium* containing a transgene cassette of interest; the mixture is exposed with the virulence agent acetosyringone, which signals the bacterium to infect wounded plant tissue. After transformation of the target cell by the bacterium, the selection is made using an antibiotic that can be selective against the bacterium while selecting the transformed cells. Variable transformation efficiencies have been reported depending on the protocol followed [103,108,109,110]. Factors such as temperature, pH, and time of virulence gene induction have been reported to have a substantial effect on transformation efficiency [111]. This method may enable integrating multigene pathways into host genomes as the capacity of the Ti plasmid has been reported as large as 150 kbp. If stably integrated into the genome, this would vastly outperform current methods of gene delivery that exhibit random nuclease digest *en route* to the nucleus, especially in green algae [112,113,114]. However, a recent report indicated that this method is no better than electroporation for stable integration of a plasmid containing a luciferase expression cassette and a selectable marker cassette into *Parachlorella kessleri* [103]. Further investigation is required to determine if this standard plant transformation protocol can be robustly applied to enhancing multi-gene expression cassette delivery to algal hosts.

## 4. Non-Traditional and Emerging Transformation Technologies

### 4.1. Cell-Penetrating Peptides

Protein vehicles for DNA transformation hold promise for enhancing transformation efforts in algae. Owing to non-covalent interactions with DNA structures, protein molecules can assist in the penetration of nucleic acids into cells and affect their integration into the genome [115]. Small delivery peptides, known as cell-penetrating peptides (CPP), have been the most commonly used among different cell-penetrating compounds. CPPs are small peptides (<30 amino acids) that can mediate the penetration of protein-cargo complexes into cells [116]. CPPs have been found from many different origins as some are derived from translocation peptide signals, and others are chimeric peptides with combined or engineered domains [117,118].

CPPs can be categorized as amphipathic, cationic, and hydrophobic, based on their physical chemistries and interaction patterns with cellular structures. Amphipathic CPPs have both hydrophobic and hydrophilic characteristics. Their amphipathic properties arise primarily from α-helical structures of both polar and non-polar amino acid regions, which are involved in their intracellular transport and preferential accumulation in the nucleus [119]. Common amphipathic peptide examples include modified poly-lactic-co-glycolic acid (MPG) peptide, transportan, tumor suppressor peptide (ARF), model amphipathic peptide (MAP), and vascular epithelial cadherin (pVEC), which have all been used to mediate cellular uptake of various substrates in cell-culture [120,121,122]. MPG has been found to have a relatively high affinity for single and double-stranded DNA as well as rapid (<1 h) and efficient (90%) delivery of fluorescent oligonucleotides into the nucleus of human fibroblast cells [120]. MAP was developed to provide a non-endocytic translocation vehicle into endothelial cells [122]. MAP is taken up by cells directly by several mechanisms: inverted micelle, pore formation, and membrane thinning [123]. Another CPP, pVEC, was used to study internalization patterns in plant epidermal and leaf cells. Cellular uptake of fluorescently labeled pVEC in plant cells was concentration and inversely temperature-dependent [121]. 

Cationic CPPs are peptides that contain a continuous chain of basic amino acids in their domains. Common cationic motifs are oligo-arginines, leucine-zipper, and paired tryptophan peptides. Cationic motifs act as CPPs due to their strong electrostatic binding and transient permeabilization of the cell membrane [124,125]. The transactivating transcription protein (Tat) and penetratin are two commonly employed cationic CPPs [126,127]. Tat has been used to demonstrate the importance of cationic motifs in CPP internalization and nuclear translocation using embryonic rat brain cell cultures [127]. Hydrophobic CPPs contain non-polar amino acids in their motifs with a high affinity for hydrophobic zones of the cell membrane [124]. However, hydrophobic CPPs have rarely been reported, and only for G protein-coupled receptor delivery, due to the complicated chemical strategies needed for their production [128]. 

It has been shown that CPP-mediated delivery systems do not have a cytotoxic effect in cyanobacteria. In *Synechocystis* sp. PCC 6803, the Tat peptide, coupled with the green fluorescence protein (GFP), was efficiently internalized by endocytosis [129]. The internalization rate may differ according to the cell type (yeast, mammalian, algae, or plant). CPPs have a predisposition to move to the nucleus after being internalized in mammalian cells, a tendency that might also be true for other eukaryotes such as algae [129,130,131]. This property necessitates viability assays for each target cell type [121,130,132,133]. Similar to other transformation methods, the presence of a cell wall is an issue for effective CPP-DNA delivery. A CPP-dsDNA complex was able to penetrate *D. salina,* which is naturally cell wall-deficient, but not into *P. tricornutum* or cell wall containing *C. reinhardtii* [130,132].

CPPs have been applied in *Drosophila antennapedia*, *Saccharomyces cerevisiae*, *Candida albicans*, human lymphocytes cells, and the cyanobacteria *Synechocystis* sp., *S. elongatus*, as well as the eukaryotic algae *D. salina*, *P. tricornutum*, *Chlorella vulgaris*, and *C. reinhardtii*. In these systems, translocation of fluorescent proteins, phosphatases, dsDNA, and dsRNA into the cells was shown [126,129,130,132,133,134,135,136]. Diverse CPPs have been used in *C. reinhardtii* to deliver fluorochrome conjugates. It was noted through flow cytometry and confocal microscopy that Tat, pVEC, penetretrin, and transportan exhibit variable performance in cargo delivery. In a cell wall containin C. reinhardtii strain, pVEC seems to have the highest efficiency with uptake at 10 µM in 15 min, similar to *S. cerevisiae* and *C. albicans* [134,135]. pVEC has also been recently demonstrated to enable ribonucleoprotein (RNP) delivery to *C. reinhardtii* with or without cell walls to knock out *Maa7* and *FKB12* genes [137]. The delivery is based on the hydrophobic region of pVEC which interacts with the external side of a cell wall and further translocates the CPP and cargo across the lipid bilayer [137,138]. However, no study has yet reported gene expression construct delivery into algal cells mediated by CPPs. 

### 4.2. Cell-Penetrating Polymers

Beyond the surfactant properties of PEG, polymers such as polyethyleneimine (PEI), polyethylene acrylic acid (PEAA), and polyamidoamine (PAMAM) have also been shown to mediate DNA delivery in the absence of exogenous endosomolytic agents to various cell types [139,140]. These polymers have been used successfully to transform mammalian cells and have been extensively studied because of their efficient endo-lysosome escape properties [141,142]. Polymer acetylation, the addition of permethyl, perethyl, choline, and long-chain alkyl groups have increased gene delivery efficiency up to 26-fold [143,144]. The advantages of polymer encapsulation of DNA are efficient protection from degradation and the possibility of polymer chemical functionalization to improve the target specificity [145]. Polymers represent a promising DNA delivery alternative in algal biotechnology due to their excellent encapsulation capability and demonstrated stabilizing properties. However, further development of polymer chemistries is needed. There are currently no reports of polymer mediated DNA transformation to algal cells and the issue of cell wall interference observed for other strategies will likely also be a hindrance to their broader application.

### 4.3. Metal-Organic Frameworks

Metal-organic frameworks (MOFs) are protein-based nanostructures that contain metal ions linked together by organic bridging ligands to form reticular structures that can also be used to assist DNA and other cargo delivery into cells. MOFs have extraordinarily high surface areas, tunable pore size, low densities, and adjustable internal surface properties [146,147,148]. Nucleic acids can be encapsulated into peptide-inorganic nanoparticles with dimensions of 4.0–7.8 nm, and ion metals of the structures allow the hybrid carriers to be transported to the nucleus [148,149,150,151]. Aminoclay is a MOF which has been used to effectively deliver DNA, polysaccharides, enzymes, and proteins into mammalian, plant, and *C. reinhardtii* cells [152,153,154,155,156,157]. Aminoclay was used for transformation of the hygromycin-B 4-O-kinase (hygromycin–B resistance gene *hph*) into cell-wall containing *C. reinhardtii, and the* nanoparticle-DNA complex was found to enable the transformation efficiency of 503 mutants with a starting cell density of 4 × 10^6^ cells mL^−1^; 500 µL of the cell culture and 500 µL of the aminoclay-DNA (i.e., DNA = 150 ng/µL; aminoclay = 100 mg/L) mixture solution were used for transformation [152]. 

A MOF subclass formed by coupling zinc cations (Zn^+2^) with methylimidazole (ZIF-8) has been effective for delivering macromolecules, including DNA, proteins, carbohydrates, and fluorescent compounds, as well as Cas9 nuclease and single-guide RNA (sgRNA) into Chinese hamster ovary (CHO) cells [147,158,159,160,161,162]. The ZIF-8 MOF showed a loading efficiency of 17% of sgRNA-Cas9 nuclease and enhanced endosomal escape by the protonation of the ZIF-8 imidazole ring release of the cargo into the cytoplasm [147]. The ZIF-8 structure can also be modified with a coating of cell membrane derivates to improve cell-type targetability, suggesting this could also be done to facilitate alga-specific chemistries [148]. MOFs exhibit unique properties such as biocompatibility, aqueous stability, excellent pH-buffering capacity, and exceptional versatility and could be further explored for gene delivery into algal systems.

### 4.4. Liposome-Mediated Transformation

Liposome-mediated transformation is a convenient method for delivering biomolecules (proteins and nucleic acids) into mammalian, bacterial, yeast, and plant protoplast cells [163,164,165,166]. Liposomes are microscopic phospholipid cationic or neutrally charged vesicles made of a concentric single lipid bilayer. Within the vesicle, cargo to be transformed can be loaded in the central aqueous compartment, including nucleotides or proteins. Liposomes have a cationic structure that facilitates nucleic acid encapsulation; they are usually composed of 1,2-dioleoyl-3-trimethylammonium propane (DOPA) or dieleoylphosphatidylethanolamine (DOPE) and coated with 1,2-distearoyl-sn-glycero-3-phosphoethanolamine-N-[polyethylene glycol] (PEG2000-DSPE) [167,168]. Liposomes are formed based on amphipathic phospholipid characteristics that are self-assembled in aqueous media into a bilayered structures. Inside the bilayer, polar groups line up to create a water-attracting surface while their lipophilic chains face each other to yield a water-free zone. Under certain conditions (e.g., shaking or heating), the phospholipid bilayers continuously enclose the dispersing aqueous medium and form a vesicular system. This structure enables encapsulation of hydrophilic and hydrophobic materials inside the inner aqueous core and the lipid bilayers, respectively. Liposome-encapsulation generates a stable complex, protecting genetic cargo from degradation outside of the cell [169]. Liposome complexes fuse with the negatively charged cell membranes and merge into cells, releasing the internal cargo [164]. These structures are interesting emerging tools for transforming multiple plasmids because they form a protective layer against enzymatic degradation outside of the cell [170]. However, lipid composition, liposome preparation, membrane bilayers (lamellarity), and sizes have to be optimized for appropriate efficiency [170,171,172]. Transformation with liposomes requires protoplasts or cell-wall deficient lines, and cargo is not protected from enzymatic digestion inside the cells upon liposome release, which is the main drawback of this technique [170]. Thus far, there are no reports of the liposome-mediated transformation of algae. However, liposomes should be useful in transforming cell-wall deficient algae strains of *C. reinhardtii*, *D. salina,* and *C. merolae* or protoplasts made from cell-wall containing species.

## 5. Considerations for the Future of Algal Transformation

The choice of the transformation method substantially impacts the overall effectiveness of an engineering strategy for a target host as this step enables the transfer of heterologous DNA into a target organism/cell line. Highlighted in this work are some transformation techniques for DNA and protein complexes that have either not yet been applied or have only recently been tested in algae, including cell-penetrating peptides, polymers, liposomes, and metal-organic-frameworks. These techniques may open new avenues of efficiency for protected DNA construct delivery to algal genomes. Whichever method is chosen for a target alga, it is essential to consider infrastructure requirements, the availability of molecular tools, expression efficiency, and reproducibility. Some of these factors are compared in Table 1. Considerations such as nuclear or organellar genome target, chromosomal/plasmid integration or episomal expression, and desired transgene copy number play a role in deciding which transformation technique to use. 

Although many examples exist, it is clear that there is no one-size-fits-all transformation strategy as each host organism has its own set of circumstances, especially related to availability of molecular tools and the presence/absence of a cell wall. Techniques such as bacterial conjugation and *Agrobacterium*-mediated transformation have some reported success in algal strains [103,104,106,107,108,109]. Conjugation has shown promise for transfer circular self-replicating episomal plasmids into diatoms and chromosomal integration in some microalgal species [79,87,92]. The major limitation in most algal systems is delivering multiple sets of transgenes in one transformation round. It remains to be seen if the non-traditional transformation techniques discussed above can help enable the stable transformation of larger DNA fragments containing multigene pathways in single transformation steps. This is especially important to reduce degradation/rearrangements and promote other avenues of genome manipulation in green algal species. 

## 6. Conclusions

In addition to reviewing some classical transformation methods for both DNA and other molecules, this work has sought to bring attention to lesser-investigated yet emerging modes of transformation to the algal biotechnologist. Perhaps these alternative techniques can enhance transformation and transgene expression efficiency rates, or yield more controlled and targeted delivery to generate high-frequency tailored genome manipulations in both model and emerging algae hosts.

## Figures and Tables

**Table 1:** Advantages and disadvantages of transformation strategies and reported efficiencies in algae and cyanobacteria.

Method	Species	Advantage	Disadvantage	Transformation Efficiency (cells/µg DNA)	Initial Cell Concentration (cells/mL)	DNA Added (µg)	Ref.
Glass bead agitation and PEG mediated DNA delivery	*C. reinhardtii*	Simple; inexpensive; fast	Requires cell wall removal/deficiency; occasional genome lesions	10^3^	10^8^	2	[14,15]
	*C. merolae*						
	*C. vulgaris*						
	*C. ellipsoidea*						
	*D. salina*						
	*protoplasts*						
Electroporation	*C. reinhardtii*	Not affected by cell wall presence; Occasional genome lesions	Specialized equipment	10^5^	10^8^	2.5	[34,37,46,48]
	*M. neglectum*						
	*Nannochloropsis* sp.						
	*P. tricornutum*						
	*Anabaena* sp.						
	*N. punctiforme*						
	*N. limnetica*						
Digital microfluidic electroporation (DME)	*C. reinhardtii*	Not affected by cell wall presence; occasional genome lesions	Specialized equipment	10^4^	10^6^	1	[49]
Square electric pulse electroporation	*C. reinhardtii*	Not affected by cell wall presence; occasional genome lesions	Specialized equipment	10^3^	10^7^	0.1	[47]
Microparticle bombardment (gene gun)	*C. reinhardtii*	Plastid target; not affected by cell wall	Cell viability compromise; specialized equipment	10^2^	10^5^	0.1	[23,51,52,53,59]
	*P. purpureum*						
	*D. salina*						
	*V. carteri*						
	*P. tricornutum*						
Natural transformation	*Anacystis nidulans*	Straightforward method for extensive genetic engineering	Limited to some species	10^4^	10^7^	5	[67,68,75]
	*Synechocystis* sp.						
	*Synechococcus* sp.						
	*T. elongatus*						
Bacterial conjugation	*Anabaena*	Low non-target insertions/knockouts; independent episome replication; allows delivery of large DNA fragments	Relies on target species characteristics based on recipient capability to integrate or maintain the vector	10^4^–10^6^	10^7^–10^9^	30–50	[81,82,84,85,86]
	*Nostoc* sp.						
	*Prochlorococcus* sp.						
	*Synechococcus* sp.						
	*Synechocystis* sp.						
	*N. punctiforme*						
	*P. tricornutum*						
	*T. pseudonana*						
	*A. obliquus*						
	*N. oleoabundans*						
	*N. oceanica*						
*Agrobacterium*-mediated transformation	*C. reinhardtii*	Low gene rearrangements; low foreign transcript silencing	Labor-intensive; no higher gene expression reported	10	10^8^	30	[103,109]
	*H. lacustris*						
	*Chlorella* sp.						
	*D. bardawil*						
	*Symbiodinium* sp.						
	*Nannochloropsis* sp.						
	*P. kessleri*						
Cell-Penetrating Peptides	*Synechocystis* sp.	High cargo stability; internalized efficiently	Requires cell wall removal/deficiency; optimized for mammalian cells	10^4^	10^5^–10^6^	10–50	[129,130,132]
	*S. elongatus*						
	*C. reinhardtii*						
	*C. vulgaris*						
	*P. tricornutum*						
	*D. salina*						
	*N. oleoabundans*						
	*S. dimorphus*						
	*Botrycoccus braunii*						
Metal-Organic Frameworks (MOF)	*C. reinhardtii*	High aqueous stability pH-buffering capacity, versatile	Not yet optimized requires cell wall removal/deficiency	10^2^	10^6^	0.7	[152]

## Data Availability

All data presented in this manuscript are sourced from the respective publications cited herein. No other experimental data were generated for the writing of this manuscript. All illustrations have been produced using BioRender.com (accessed on 31 January 2021).
